# Hybrid Osteosynthesis for Septic Humeral Shaft Nonunion in a Patient with Obesity: A Case Report

**DOI:** 10.7759/cureus.119283

**Published:** 2026-11-07

**Authors:** Paolo Chiquini-Ramírez, Nancy Lorena Villamues-Moreno, Jorge Ponce-de-Léon-Dominguez, Leonardo Moreno-Degante, Bernardo Pettet-Ruiz, Ernesto Torres-Casado, Héctor Mauricio Reyes-García, Nelva Garduza-Leyva

**Affiliations:** 1 Department of Orthopedic Surgery and Traumatology, Instituto Nacional de Rehabilitación Luis Guillermo Ibarra Ibarra, Mexico City, MEX; 2 Department of Orthopedic Surgery and Traumatology, Centro Médico ABC, Mexico City, MEX; 3 Department of Orthopedic Surgery and Traumatology, Instituto Nacional de Rehabilitacion Luis Guillermo Ibarra Ibarra, Mexico City, MEX

**Keywords:** dynamic compression plate, humeral shaft, hybrid osteosynthesis, intramedullary nail, obesity, septic nonunion, staphylococcus epidermidis

## Abstract

Septic humeral shaft nonunion represents a complex reconstructive challenge in orthopedic surgery, particularly in patients with obesity, in whom increased lever arms and greater mechanical loading of the upper extremity substantially compromise fixation stability. Recurrent failure of isolated fixation constructs, including intramedullary nails or compression plates, necessitates hybrid strategies that combine enhanced biomechanical stability with effective eradication of microbial infection.

This article presents the case of a middle-aged man with obesity and a history of a left humeral shaft fracture initially treated with intramedullary nailing in December 2020 who developed persistent nonunion. Following revision surgery with dynamic compression plating, fixation failure occurred secondary to implant fatigue. A second revision procedure was performed through a posterior approach, including nerve neurolysis, extensive debridement, and hybrid fixation using a 240 x 8 mm Targon™ H intramedullary nail (B. Braun, Melsungen, Germany) combined with a narrow 4.5-mm dynamic compression plate (DCP; DePuy Synthes, Warsaw, IN, USA). Cancellous bone chip grafting was also performed. Intraoperative cultures yielded *Staphylococcus epidermidis, *establishing the diagnosis of septic nonunion, and targeted oral clindamycin therapy was initiated. Despite transient postoperative radial neuropraxia, which resolved with pregabalin treatment and structured physical rehabilitation, the patient achieved complete osseous union, eradication of infection, and an Upper Extremity Functional Scale (UEFS) score of 69/80, corresponding to 86% of maximal functional capacity.

In conclusion, hybrid fixation combining an intramedullary nail with a narrow 4.5-mm DCP, together with pathogen-directed antimicrobial therapy, provides excellent axial and rotational stability and represents an effective salvage strategy for septic humeral shaft nonunion in patients with increased biomechanical demands.

## Introduction

Humeral shaft fractures account for approximately 3% to 5% of all appendicular skeletal fractures. Although most cases respond favorably to either conservative management or primary surgical fixation, nonunion remains a significant complication, with reported rates ranging from 2% to 15%. The risk increases substantially following high-energy trauma, severe comminution, short transverse fracture patterns, and in patients with systemic comorbidities such as obesity, which subjects the humeral shaft to abnormal mechanical stresses during routine daily activities [1-3].

When chronic mechanical instability is compounded by bacterial colonization and biofilm formation, the clinical entity evolves into septic nonunion, also referred to as fracture-related infection (FRI). This condition represents the simultaneous failure of both the biological and mechanical environments required for fracture healing. Local tissue ischemia, microvascular necrosis, depletion of osteogenic potential, and inadequate construct rigidity collectively impair bone regeneration. Low-virulence microorganisms, particularly *Staphylococcus epidermidis*, frequently evade initial clinical detection and may present solely as repeated implant failure caused by fatigue rather than with overt signs of infection [3-6].

Successful management of refractory septic humeral shaft nonunion requires strict adherence to the principles of the Diamond Concept. These include radial debridement of all devitalized tissue, restoration of osteoconductive scaffolding and osteoinductive biological stimuli, optimization of microbial control, and, critically, the establishment of absolute mechanical stability [7,8].

Traditionally, revision surgery using a single compression plate has been the preferred reconstructive technique. However, in the presence of sclerotic bone, widened intramedullary canals, cortical defects, or obesity, isolated plate fixation is subjected to continuous cantilever bending forces that frequently result in screw loosening or implant fatigue. Conversely, exchange intramedullary nailing alone often fails to provide sufficient rotational control or micromotion at the site of atrophic nonunion [9-11].

To overcome these biomechanical limitations, hybrid osteosynthesis combining an intramedullary fixation with the rotational stability and interfragmentary compression provided by plate fixation, this hybrid construct offers superior biomechanical performance compared with either technique alone [12,13].

The purpose of this report is to describe the successful management of refractory septic nonunion of the left humeral shaft in an obese patient who had undergone multiple previous unsuccessful surgical procedures. Treatment consisted of hybrid osteosynthesis using Targon™ H intramedullary nail (B. Braun, Melsungen, Germany) supplemented with a narrow 4.5-mm dynamic compression plate (DCP), followed by a review of the relevant literature [14-17].

## Case presentation

A 53-year-old man presented with a medical history significant for Class III obesity (body mass index (BMI), 52.9 kg/m^2^), secondary hypertension, obstructive sleep apnea syndrome, otosclerosis, a 20-year smoking history (smoking index: 18 pack-years), and documented penicillin allergy.

The patient’s current condition originated in November 2020, following a motorcycle collision. He was struck on his right side by a moving vehicle, causing him to be thrown from the motorcycle and sustain direct impact of his left arm against the pavement. He immediately developed pain, visible deformity, crepitus, and functional impairment of the affected extremity. He was transferred to a trauma and orthopedic referral center, where clinical evaluation and radiographic studies established the diagnosis of a left humeral shaft fracture classified as AO Foundation/Orthopaedic Trauma Association (AO/OTA) type 12A3b (Figure 1).

**Figure 1:**
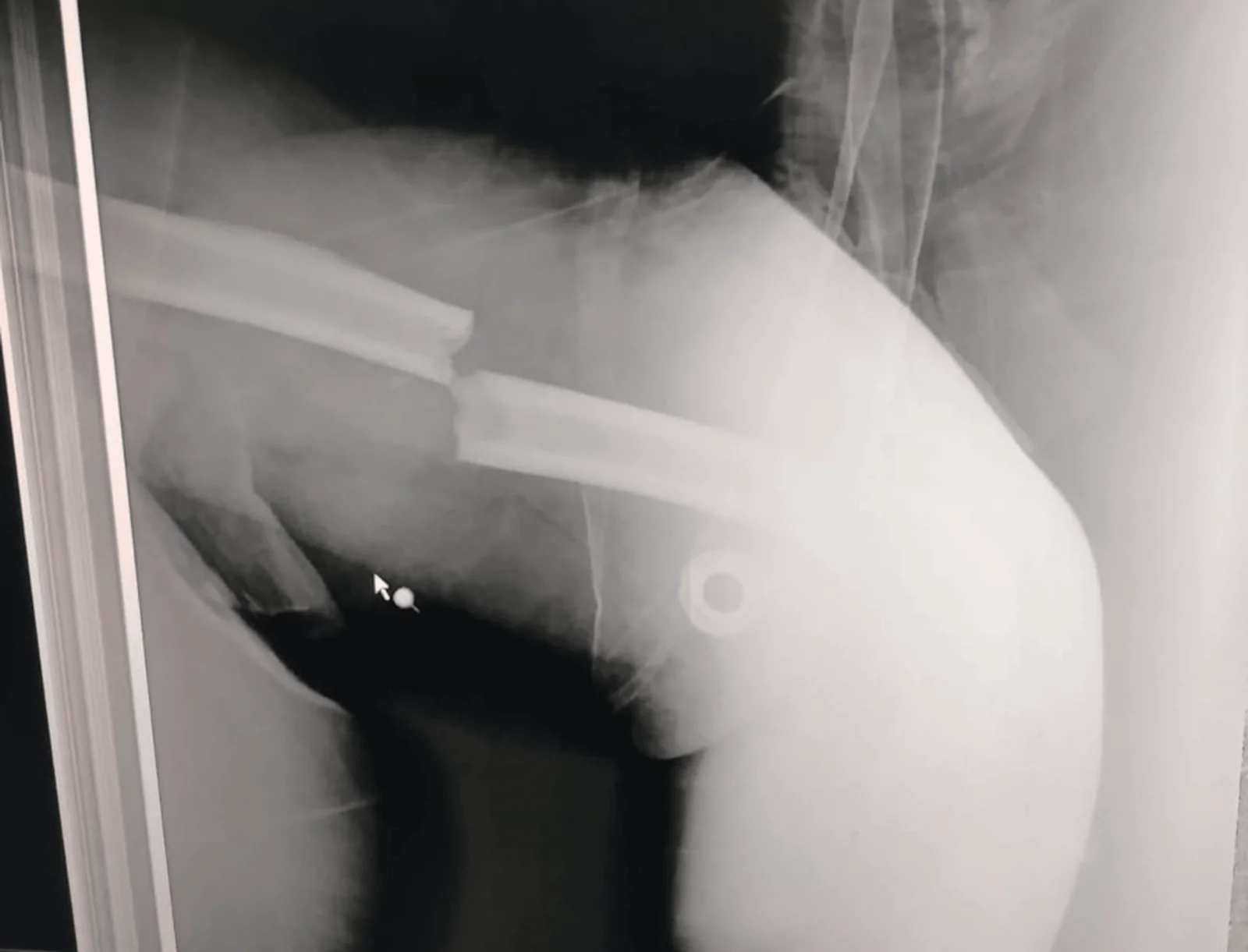
Initial lateral radiograph of the left humerus.

In December 2020, he underwent closed reduction and internal fixation with an intramedullary nail (Figure 2). 

**Figure 2:**
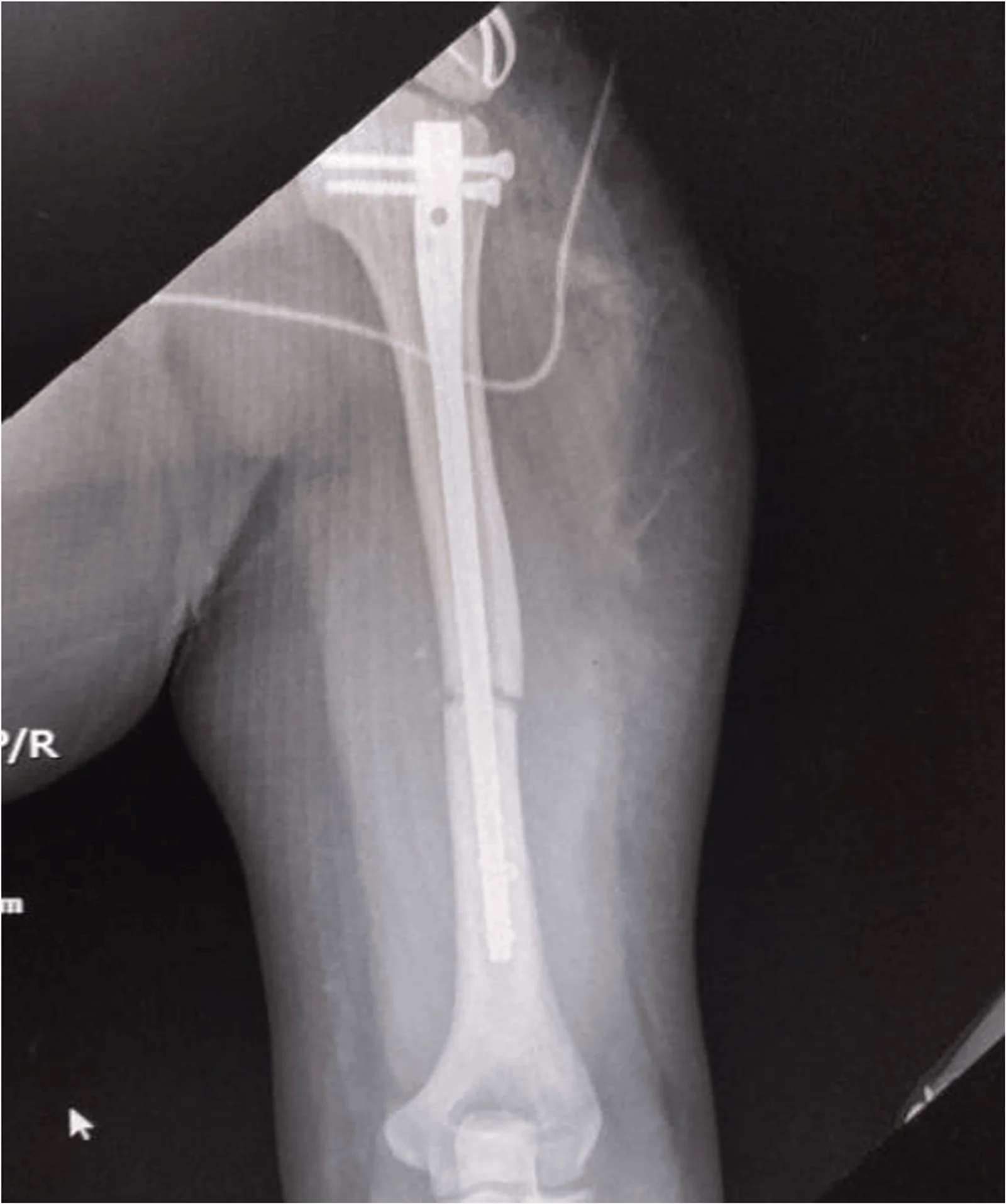
Postoperative anteroposterior radiograph of the left humerus obtained on December 2020, following treatment with an intramedullary nail.

At routine follow-up one year after surgery, the patient reported persistent pain in the left arm during physical exertion. Radiographs demonstrated nonunion of the left humeral shaft without evidence of implant fatigue (Figure 3).

**Figure 3:**
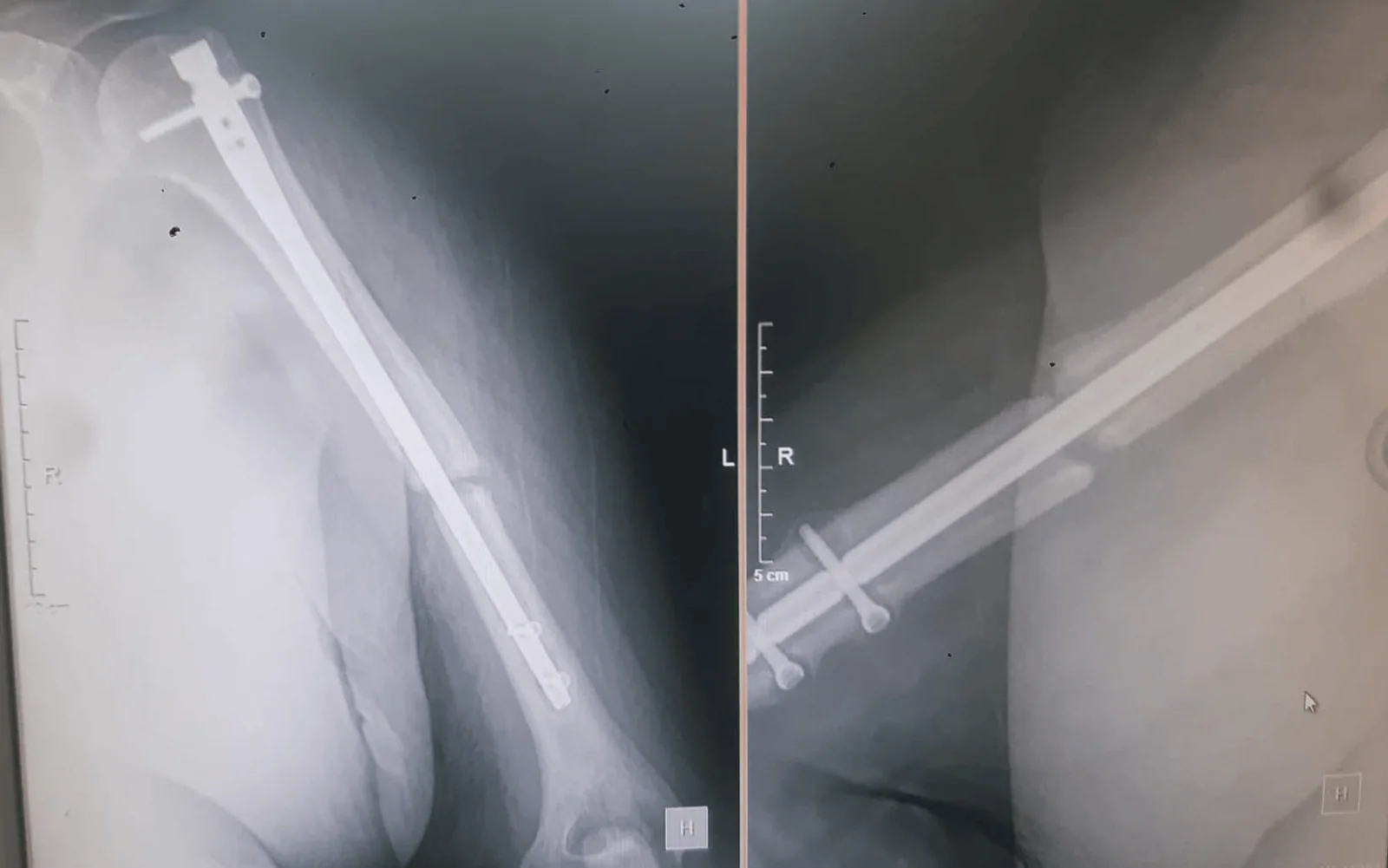
Anteroposterior and lateral radiographs of the left humerus obtained one year after surgery, demonstrating humeral nonunion without evidence of intramedullary nail fatigue.

Clinical examination revealed a well-healed surgical incision without signs of infection. Based on these findings, revision surgery was scheduled at the treating institution, and in December 2021, the intramedullary nail was removed, the nonunion site was explored, and revision fixation was performed using a lateral dynamic compression plate (Figure 4).

**Figure 4:**
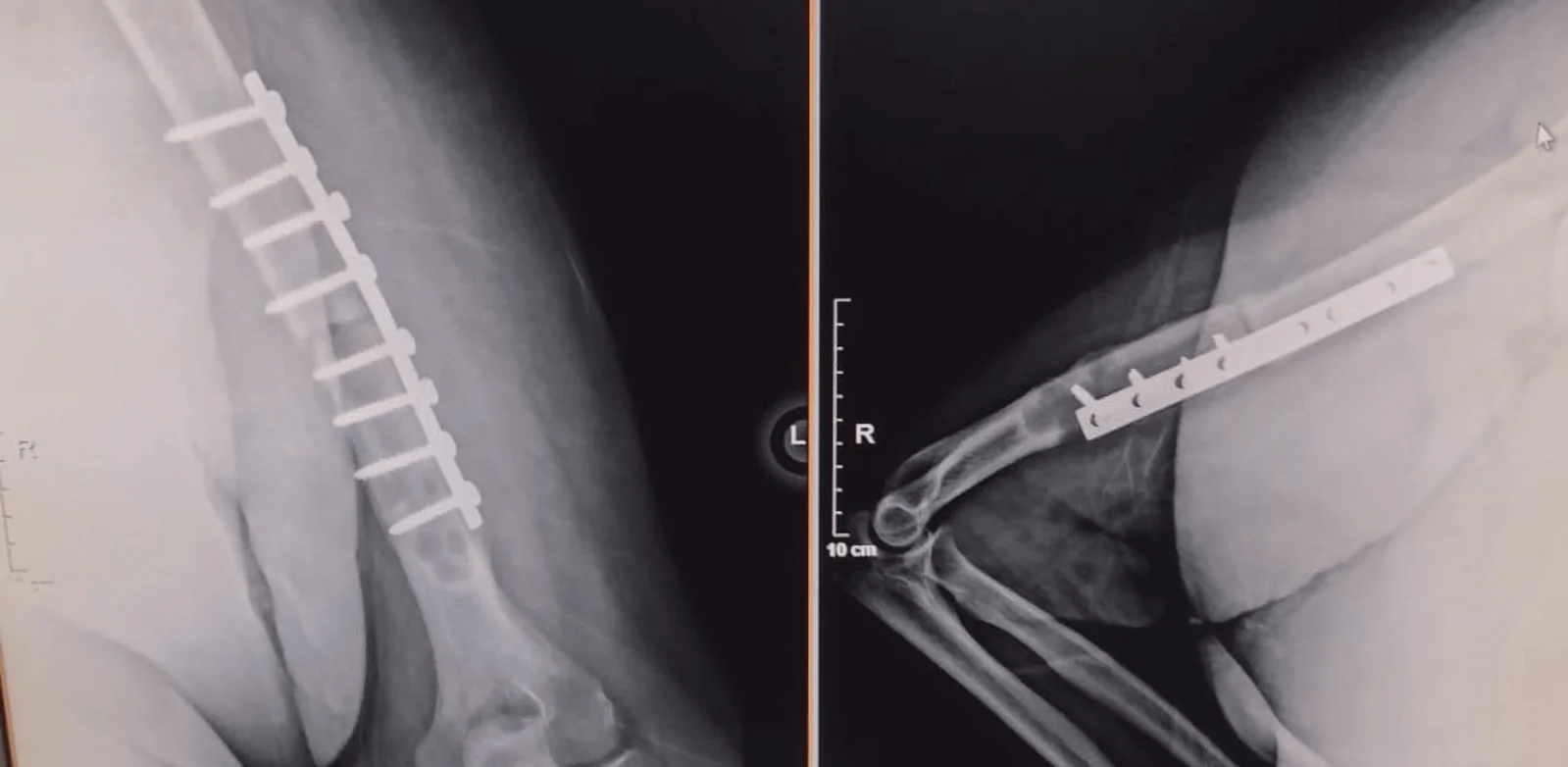
Anteroposterior and lateral radiographs of the left humerus obtained during revision surgery of left humeral nonunion, demonstrating fixation with a dynamic compression plate.

At a subsequent follow-up visit in March 2022, radiographs continued to demonstrate absence of osseous union, although no evidence of hardware fatigue was observed (Figure 5).

**Figure 5:**
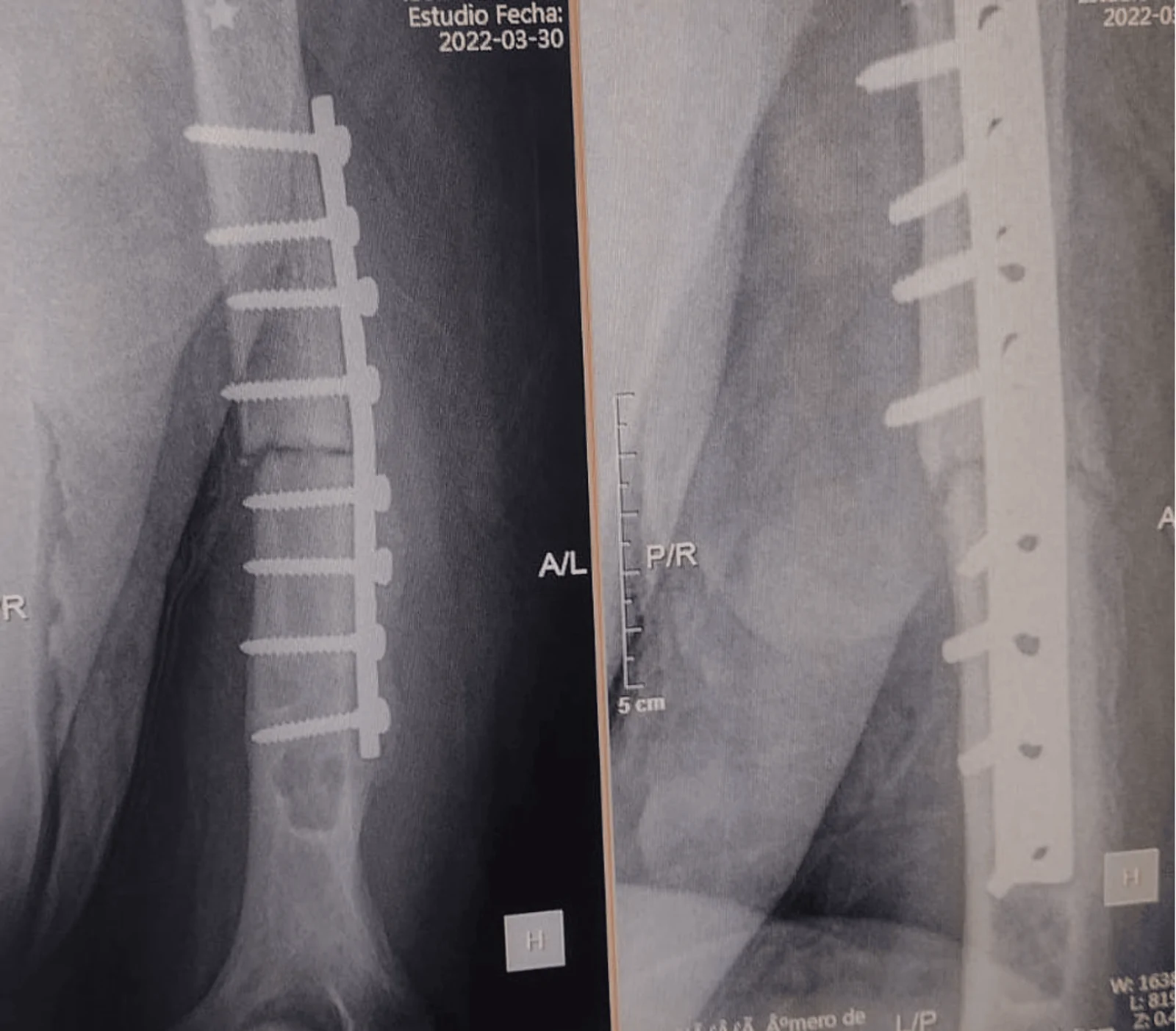
Anteroposterior and lateral radiographs of the left humerus demonstrating no evidence of osseous union and no evidence of hardware fatigue.

However, in June 2022, following physical exertion, the patient experienced sudden crepitus in the left arm associated with acute pain and complete loss of function. He presented to the emergency department of the treating hospital, where radiographs confirmed persistent humeral shaft nonunion accompanied by fatigue failure of the fixation construct at the level of the nonunion (Figure 6).

**Figure 6:**
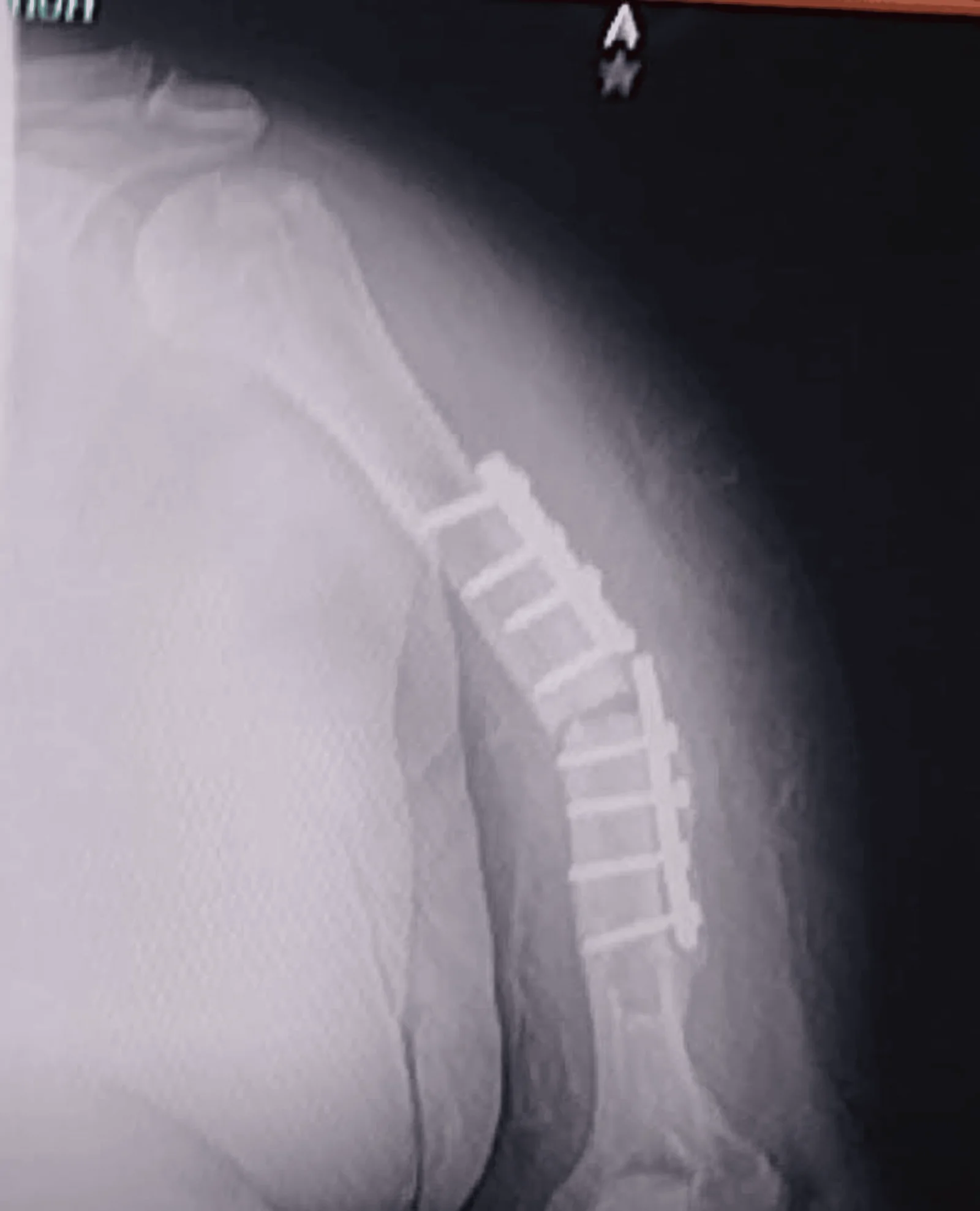
Anteroposterior radiograph of the left humerus demonstrating nonunion of the humeral shaft with hardware fatigue.

Seeking further treatment, the patient was evaluated in July 2022 at the Department of Orthopedic Trauma of the Luis Guillermo Ibarra Ibarra National Institute of Rehabilitation, Mexico City, Mexico. Physical examination revealed a well-healed surgical scar without erythema, warmth, drainage, or other clinical signs of infection. Palpation elicited pain and crepitus over the midshaft of the humerus. Shoulder and elbow range of motion could not be reliably assessed because of pain, whereas distal neurovascular examination was unremarkable.

Computed tomography (CT) of the left arm precisely identified the nonunion site within the midshaft of the humerus (Figure 7).

**Figure 7:**
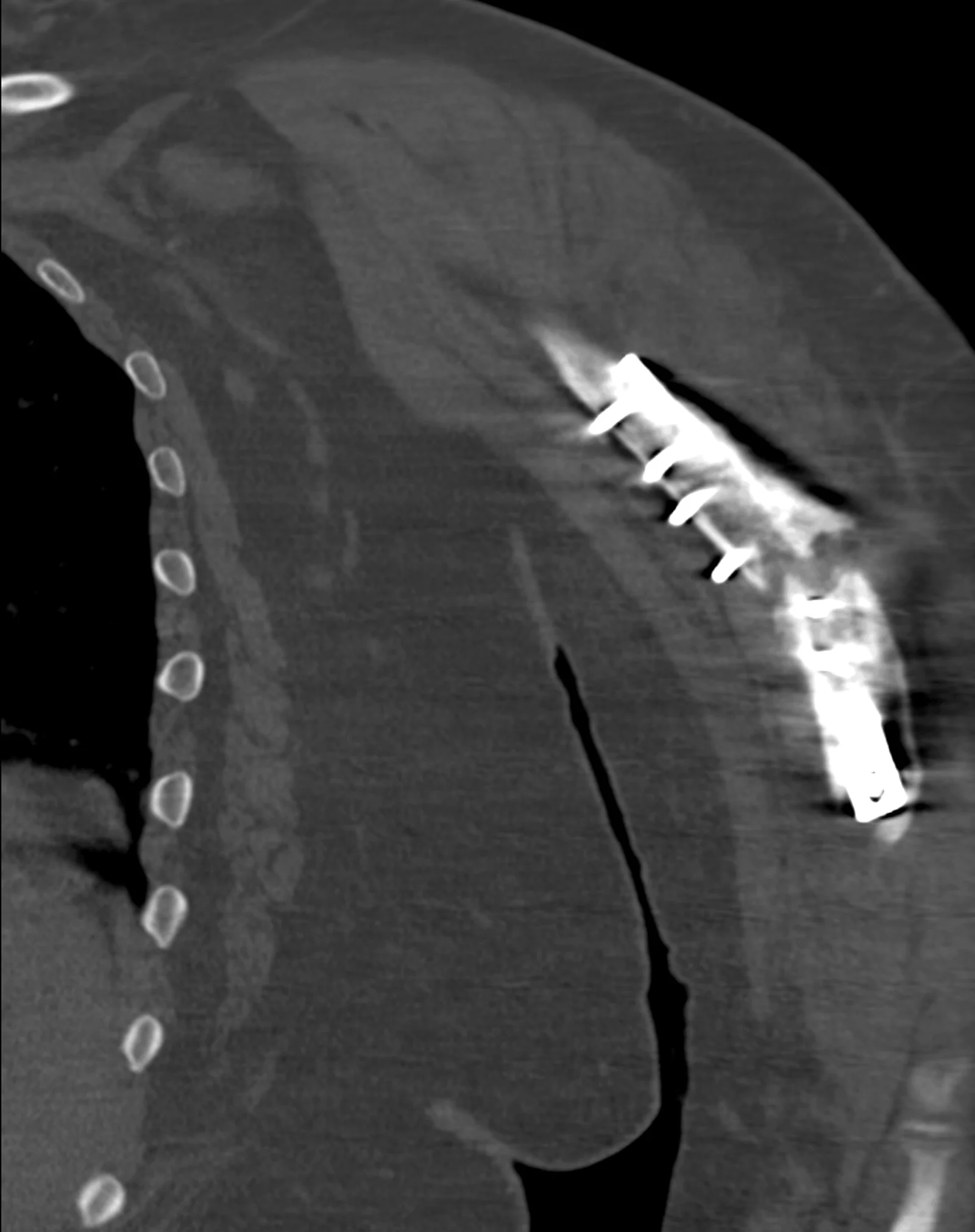
Coronal computed tomography image of the left humerus demonstrating a nonunion site in the midshaft of the left humerus with hardware fatigue.

Laboratory investigations demonstrated a white blood cell count of 5.9 x 10³/µl, hemoglobin concentration of 17.2 g/dL, erythrocyte sedimentation rate of 12 mm/h, and high-sensitivity C-reactive protein level of 0.30 mg/dL; laboratory findings supported an initial diagnosis of aseptic midshaft humeral nonunion, and revision surgery was scheduled.

After completion of the preoperative evaluation, revision surgery was performed in December 2022, through a posterior trans-tricipital approach. The procedure consisted of meticulous identification and neurolysis of the radial nerve, complete removal of the failed fixation construct (3.5-mm DCP plate), and it was sent to the Infectious Diseases Department for sonication. No additional intraoperative tissue samples were collected during the procedure. Upon inspection of the nonunion site, a small amount of purulent discharge was observed at the nonunion focus and around the implant. Radical debridement of the nonunion site, including extensive debridement of all devitalized and compromised tissue, was performed, followed by intramedullary reaming of the humerus, which yielded tissue with purulent characteristics. Following debridement, the surgical site showed no macroscopic evidence of infection, with the remaining tissue exhibiting satisfactory characteristics according to the Scully criteria; hybrid osteosynthesis was performed using a 240 × 8 Targon™ H intramedullary nail, supplemented with a narrow 4.5-mm dynamic compression plate (DePuy Synthes, Warsaw, IN, USA) (Figure 8).

**Figure 8:**
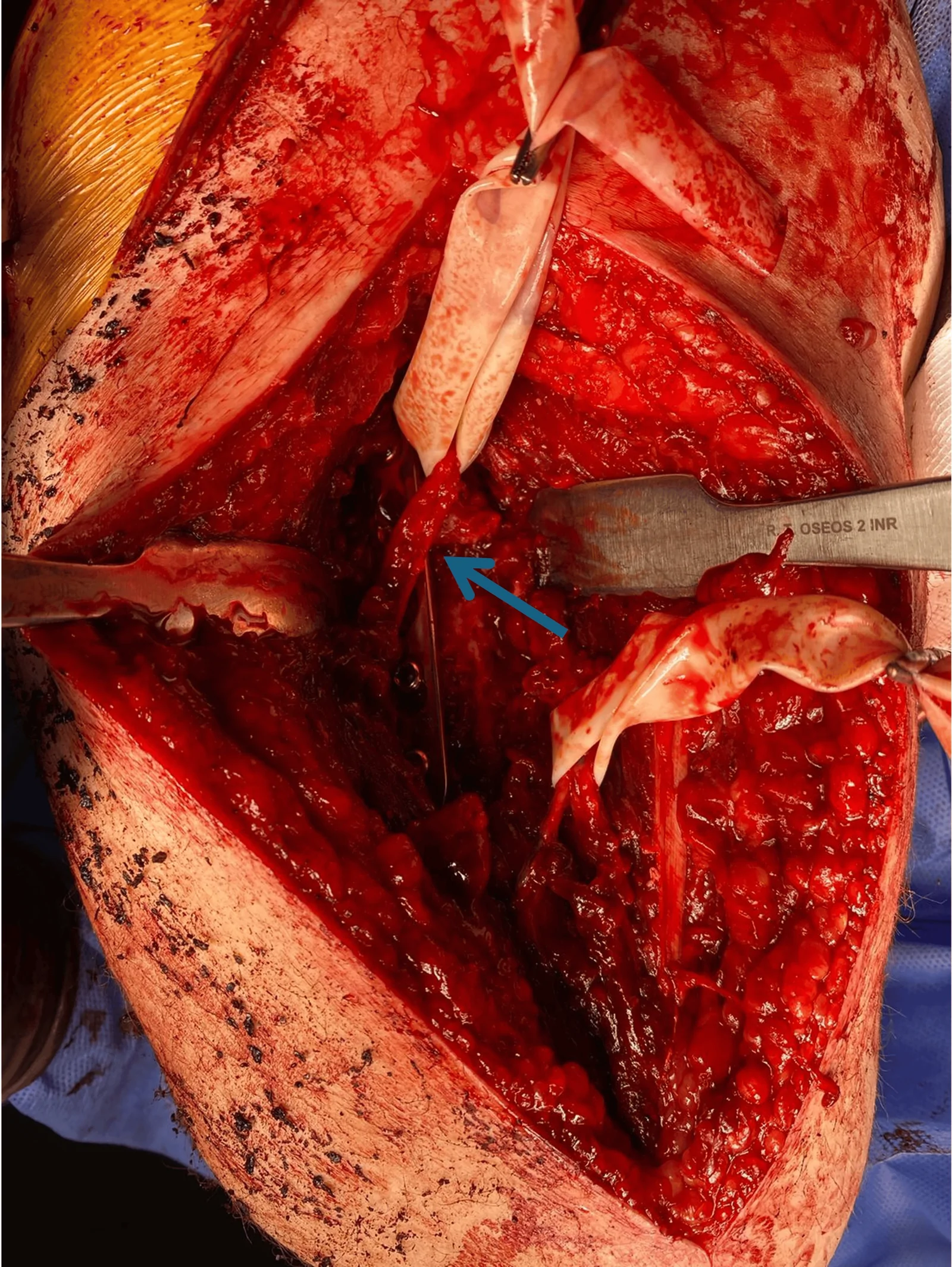
Posterior approach to the left arm demonstrating the course of the radial nerve from cephalad to caudal and from posterior to anterior.

The reconstruction was further augmented with 10 cc of cancellous bone chip allograft (Figure 9).

**Figure 9:**
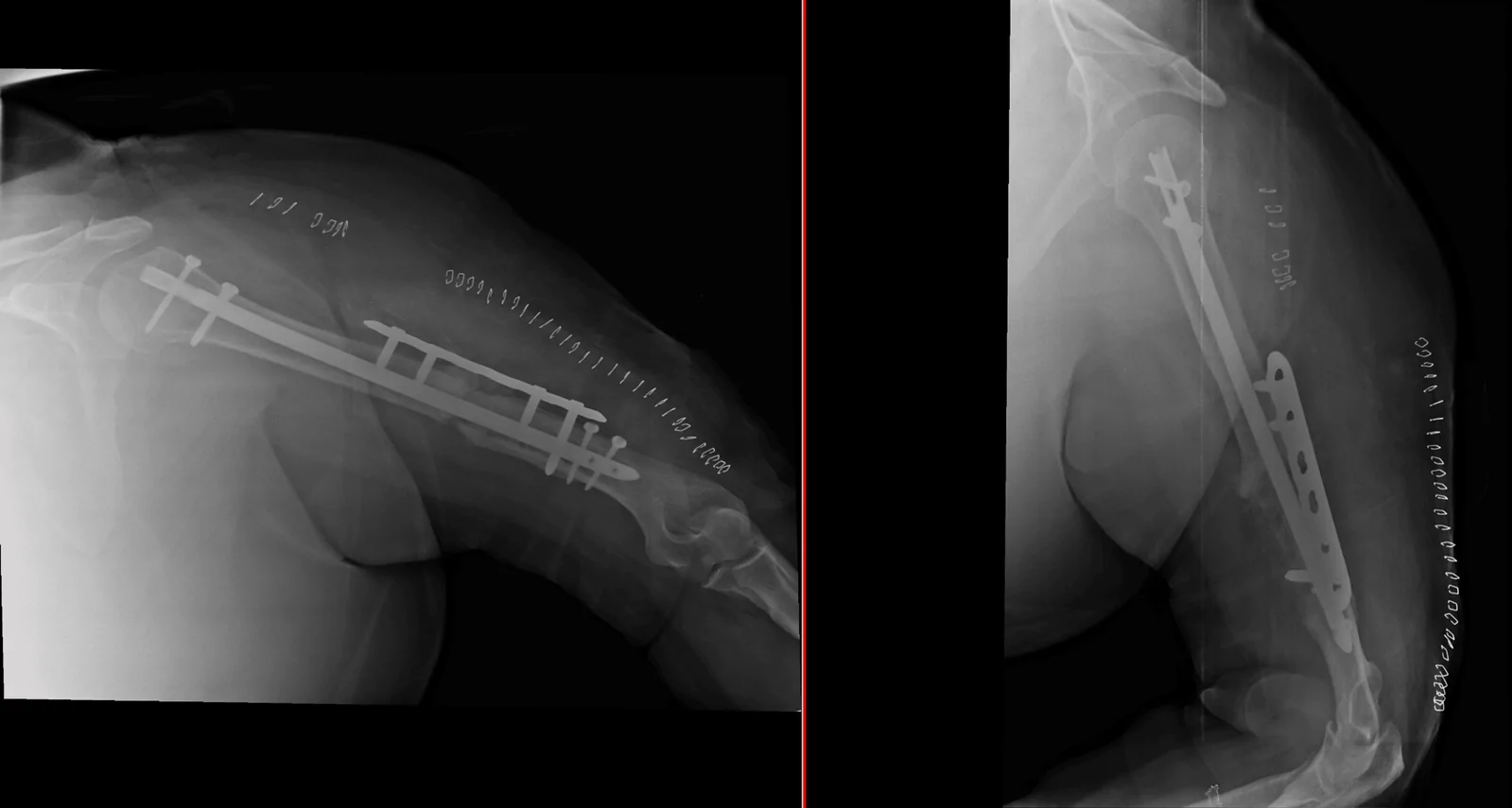
Immediate postoperative anteroposterior and lateral radiographs.

During the immediate postoperative period, the patient developed paresthesia within the C6 dermatome and weakness of wrist and finger extension, graded as 2/5 according to the Daniels Muscle Strength Scale. Treatment consisted of structured physical rehabilitation together with pregabalin (150 mg orally once daily for one month).

The sonication culture result of the osteosynthesis material yielded a positive culture for *Staphylococcus epidermidis*, susceptible to clindamycin. Based on the sonication culture results, the intraoperative findings, and the history of previous failed surgical procedures, the diagnosis was revised to septic nonunion of the left humeral shaft, and pathogen-directed antimicrobial therapy was initiated with oral clindamycin (600 mg every eight hours for seven days). Given the favorable postoperative clinical course, with no signs of persistent or recurrent infection, no further antibiotic treatment was administered.

The diagnosis of septic nonunion was established based on the combination of microbiological, intraoperative, and clinical findings. The sonication culture of the previously failed osteosynthesis hardware yielded *Staphylococcus epidermidis*. In addition, intraoperative exploration revealed purulent discharge at the nonunion site and around the implant, together with compromised tissue conditions. Furthermore, the patient had a history of two previous unsuccessful surgical procedures performed at another public healthcare institution. Considering that these procedures had apparently provided adequate fixation and mechanical stability, the repeated failure of bone healing raised a strong suspicion of an underlying infectious process contributing to the persistence of nonunion. The subsequent achievement of bone union following surgical debridement and revision fixation further supports the successful management of this complex case.

The patient’s postoperative course was highly favorable, with complete recovery of distal motor strength and sensory function. At the most recent follow-up evaluation in June 2026, the surgical wound had healed uneventfully, without clinical evidence of recurrent infection. Full range of motion of the shoulder, elbow, wrist, and hand had been restored, and no tenderness was elicited on palpation (Figure 10).

**Figure 10:**
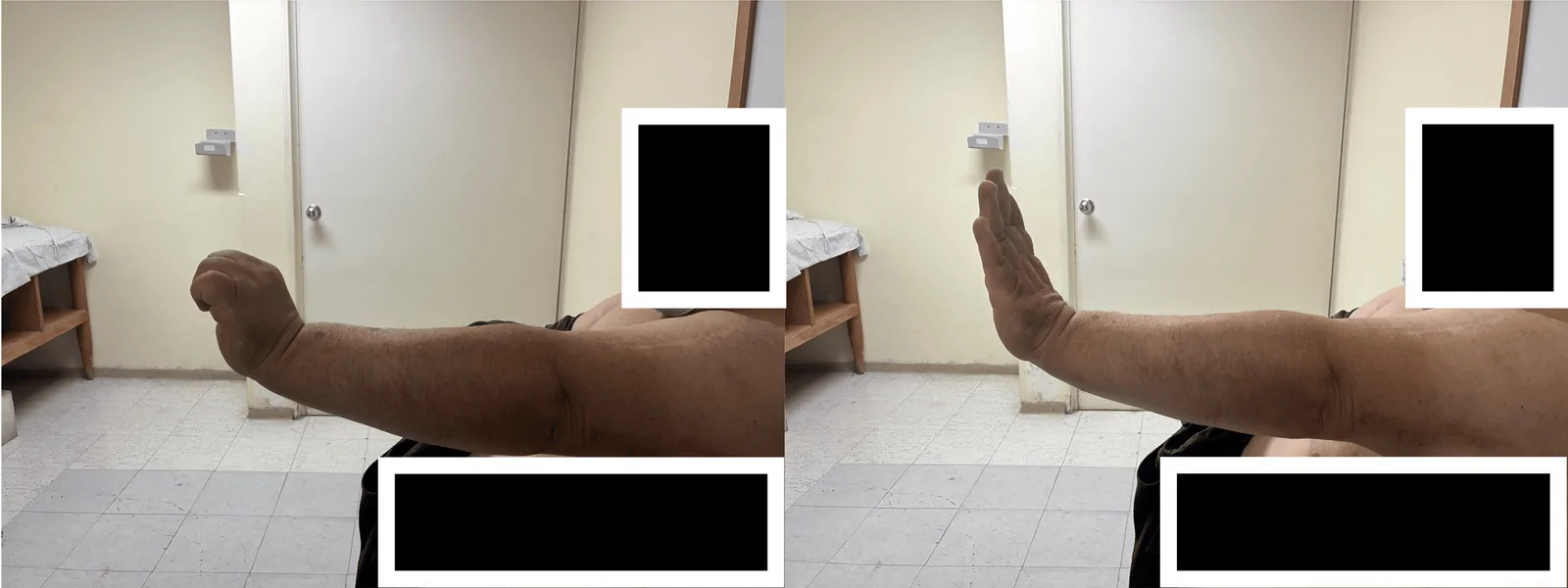
Clinical image of the patient demonstrating extension of the left wrist and hand.

Radiographs confirmed complete osseous union without evidence of implant loosening or fatigue (Figure 11).

**Figure 11:**
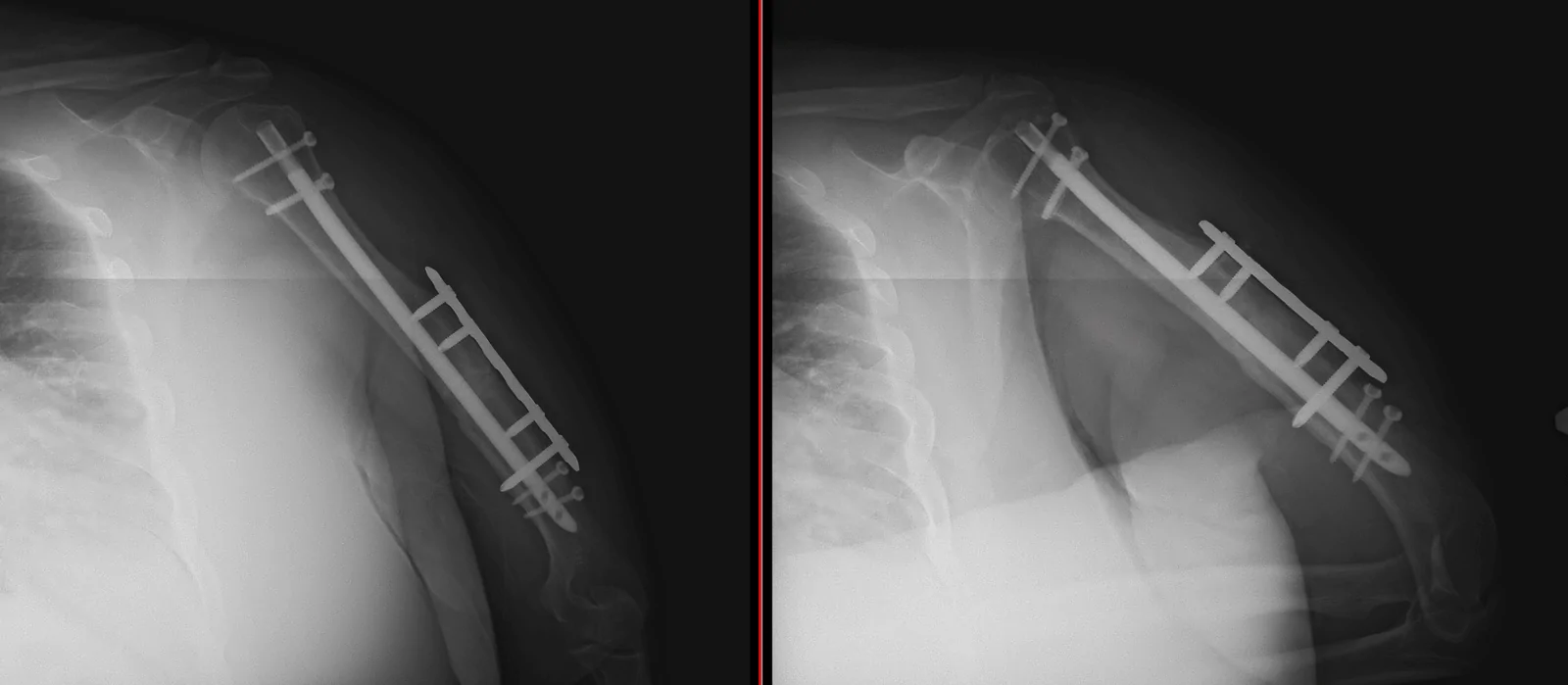
Follow-up radiograph demonstrating evidence of osseous union of the left humeral shaft.

Functional assessment using the Upper Extremity Functional Scale (UEFS) yielded a score of 69 out of 80 points (86% of maximum function), indicating only mild residual functional limitation of the affected upper extremity following reconstruction [18,19].

## Discussion

Management of refractory septic humeral shaft nonunion in a patient with obesity represents one of the most demanding challenges in contemporary orthopedic trauma surgery. The coexistence of chronic mechanical instability, previous implant failure, biofilm-producing microorganisms, and the increased biomechanical loading associated with obesity creates an exceptionally unfavorable environment for fracture healing.

Septic nonunion and obesity

FRIs caused by low-virulence pathogens such as *Staphylococcus epidermidis* frequently occur in the absence of the clinical manifestations of infection, including erythema, warmth, or purulent drainage. The organism’s ability to produce an extracellular polysaccharide biofilm on metallic implants disrupts local microvascular circulation while impairing both osteoblastic activity and host phagocytic function. This persistent biological deterioration results in continuous micromotion at the fracture site, ultimately leading to implant fatigue and mechanical failure, as observed in the present case following failure of the initial dynamic compression plate [3,5].

Obesity further compounds this process by substantially increasing the biomechanical demands placed upon the upper extremity. Patients with a markedly elevated BMI subject the humerus to considerably greater loads not only during routine daily activities but also while using the upper limbs for support during transfers or rising from a seated position. These increased loading conditions generate significantly higher bending and torsional moments across the humeral shaft, predisposing isolated fixation constructs to fatigue failure when fracture healing is delayed.

Biomechanical rationale for hybrid osteosynthesis

The principal biomechanical advantage of hybrid osteosynthesis lies in its ability to simultaneously neutralize the bending, shear, and torsional forces acting across the humeral shaft.

The Targon™ H intramedullary nail (8 x 240 mm), positioned along the neutral mechanical axis of the humerus, functions as a load-sharing device. Its central location provides excellent resistance to anteroposterior and mediolateral bending forces while reducing stress concentration along the cortical bone. Nevertheless, intramedullary fixation alone may provide inadequate rotational stability, particularly in previous instrumented or widened medullary canals.

Conversely, the narrow 4.5-mm DCP functions primarily as a rotational neutralization device. Dynamic compression across the nonunion site minimizes interfragmentary motion while restoring rigid axial stability. Experimental biomechanical investigations and finite-element analyses have demonstrated that supplementing an intramedullary nail with a compression plate increases torsional stiffness by more than 60% compared with isolated intramedullary fixation. Such construct rigidity is biologically essential because revascularization and new bone formation cannot occur when interfragmentary motion exceeds approximately 150 µm [6,9,11].

Surgical technique and the diamond concept

From a technical standpoint, successful execution of hybrid osteosynthesis in a previously operated humerus requires meticulous surgical planning. Tangential or eccentrically directed plate screws permit secure bicortical fixation while avoiding collision with the intramedullary nail. In the present case, the use of a narrow 4.5-mm DCP minimized implant bulk and limited additional periosteal stripping, thereby preserving the remaining periosteal blood supply.

Equally important was strict adherence to the principles of the Diamond Concept, which constituted the biological foundation of reconstruction. Mechanical stability was achieved through the hybrid construct consisting of the Targon™ H intramedullary nail and supplemental DCP plate. Biological optimization and debridement, radical excision of infected fibrous tissue until punctate cortical bleeding (“paprika sign”) was achieved, combined with endosteal reaming to stimulate osteogenesis. Bone grafting, placement of cancellous bone graft to provide an osteoconductive scaffold supporting cellular adhesion and new bone formation. Microbiological control, pathogen-directed antimicrobial therapy with oral clindamycin following identification of *Staphylococcus epidermidis* in deep intraoperative cultures.

The favorable outcome observed in this patient highlights the importance of integrating these biological and mechanical principles into a comprehensive reconstructive strategy.

Postoperative neurological complications

Radial nerve neuropraxia remains a recognized complication of revision surgery performed through a posterior approach to the humerus, with reported incidences ranging from 15% to 20% in repeat procedures because of dense perineural fibrosis. In the present case, systematic identification and careful neurolysis of the radial nerve throughout its course successfully prevented irreversible iatrogenic neurotmesis [17].

The transient postoperative motor and sensory deficits responded well to pregabalin therapy and early rehabilitation, resulting in complete recovery of muscle strength (Medical Research Council grade 5/5) and restoration of normal neurological function. This excellent neurological recovery was paralleled by a favorable functional outcome, as reflected by the patient’s Upper Extremity Functional Scale score of 69/80 (86% of maximum function) [18,19].

## Conclusions

Hybrid osteosynthesis combining a Targon™ H intramedullary nail with a narrow 4.5 mm DCP, complemented by thorough debridement, radial nerve neurolysis, bone grafting, and pathogen-directed antimicrobial therapy, proved to be an effective salvage strategy for the management of septic humeral shaft non-union in this patient.

The hybrid construct provided enhanced resistance to both bending and torsional forces, thereby creating a biomechanical environment conducive to fracture healing despite the increased mechanical demands associated with severe obesity. In conjunction with meticulous biological optimization and targeted infection control, this strategy resulted in successful osseous union, eradication of infection, recovery of neurological function, and an excellent functional outcome.

This case highlights the value of integrating sound biomechanical principles with biological tenets of the Diamond Concept when treating complex FRIs. Although additional clinical studies are needed to establish the reproducibility of this approach, hybrid osteosynthesis may represent a valuable reconstructive option for selected patients with refractory septic humeral shaft nonunion following failed fixation.
